# Spontaneous Resolution of Strangulated Small Bowel Obstruction in a Patient With Intestinal Tuberculosis After Starting Anti-tuberculosis Drugs: A Case Report

**DOI:** 10.7759/cureus.31229

**Published:** 2022-11-08

**Authors:** Nagisa Nakagawa, Hidesato Odaka, Kenjiro Yoshikawa, Toshiya Sawada, Hideaki Ishida

**Affiliations:** 1 Department of Medicine, Japanese Red Cross Akita Hospital, Akita, JPN; 2 Department of Respiratory Medicine, Japanese Red Cross Akita Hospital, Akita, JPN; 3 Department of Gastroenterology, Japanese Red Cross AKita Hospital, Akita, JPN; 4 Department of Surgery, Japanese Red Cross Akita Hospital, Akita, JPN; 5 Department of Gastroenterology and Hepatology, Japanese Red Cross Akita Hospital, Akita, JPN

**Keywords:** strangulated small bowel obstruction, anti-tuberculosis drugs, abdominal ultrasonography, tuberculosis peritonitis, spontaneous resolving, intestinal tuberculosis

## Abstract

Intestinal tuberculosis can cause strangulated small bowel obstruction. Strangulated small bowel obstruction usually requires surgery. We report a case of a patient with intestinal tuberculosis, who developed a spontaneously resolving strangulated small bowel obstruction after the commencement of anti-tuberculosis drugs. A 72-year-old woman presented with abdominal pain and ascites was noticed on abdominal ultrasonography. Contrast-enhanced computed tomography (CT) revealed a 50-mm tumor in the ileocecal region that was darkly contrasted, along with peritoneal thickening and ascites. A malignant tumor and carcinomatous peritonitis were suspected. Colonoscopy showed an ulcerative lesion in the terminal ileum, and the acid-fast bacillus culture was positive; therefore, the patient was diagnosed with intestinal tuberculosis and was treated with isoniazid, rifampicin, ethambutol, and pyrazinamide. After commencing treatment, improvement in peritoneal thickening and ascites was confirmed using abdominal ultrasonography; therefore, we concluded that the ascites was due to tuberculous peritonitis. Six weeks after the initiation of treatment, the patient visited our facility with complaints of abdominal pain. Contrast-enhanced CT revealed unenhanced small intestinal walls, and a diagnosis of strangulated small bowel obstruction was made; however, her symptoms improved naturally. Strangulated small bowel obstruction was presumed to be due to the presence of bands as anti-tuberculosis therapy could promote fibrosis. In this case, abdominal ultrasonography was useful in the evaluation of the effects of treatment.

## Introduction

In 2021, according to statistics from the Ministry of Health, Labor and Welfare, Japan finally became one of the countries with a low incidence of tuberculosis [1], similar to Europe and the United States. Hence, there have been fewer opportunities to diagnose and treat tuberculosis in Japan, especially cases of intestinal tuberculosis. Intestinal tuberculous can result from swallowing infected sputum, ingestion of contaminated food, hematogenous spread, or direct extension from adjacent organs [2]. The ileocecal and jejunoileal areas are the most common sites of involvement. Complications of intestinal tuberculosis include obstruction, perforation, and fistula formation [3]. Cases of strangulated small bowel obstruction could also occur. Strangulated small bowel obstruction usually requires urgent surgical treatment [4]. Herein, we report a case of a patient with intestinal tuberculosis, who developed a strangulated small bowel obstruction that spontaneously resolved after the commencement of anti-tuberculosis drugs. Abdominal ultrasonography was useful in evaluating therapeutic effects. This article was previously presented as a meeting abstract at the 145^th^ Japanese Society for Tuberculosis and Nontuberculous Mycobacteriosis, Tohoku Branch Meeting on September 10, 2022.

## Case presentation

**Figure 1:**
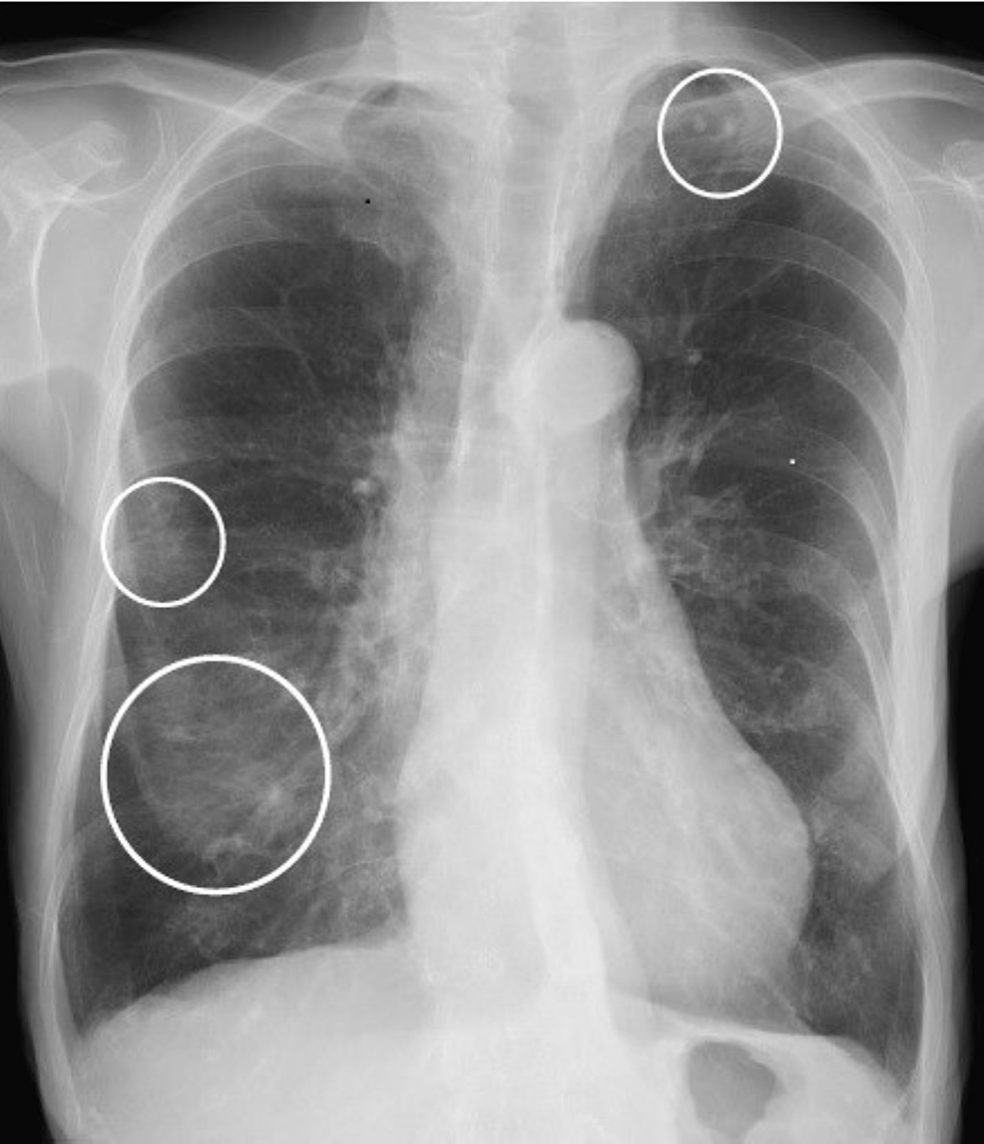
Chest X-ray scan obtained at the first visit A chest X-ray scan was obtained at the first visit, showing opacities in the right middle and lower lung fields and a granular shadow in the left lung apex.

A 72-year-old woman with no significant medical history presented with abdominal pain and distention in July 2021. She visited a local physician and abdominal ultrasonography revealed the presence of ascites; therefore, she was referred to our department for further examination and treatment. She previously smoked 12 cigarettes per day for 40 years (from 20 to 60 years of age). Her family history was significant for stomach and colon cancer in her father. At the first visit, her vital parameters were as follows: blood pressure of 148/73 mmHg, pulse rate of 66 beats/min, respiratory rate of 20 breaths/min, O_2_ saturation of 98% on room air, and temperature of 36.6°C. There were no pulmonary vessel murmurs, abdominal vascular murmurs, or other remarkable physical findings. Abnormal laboratory findings at the first visit were as follows: cancer antigen 125 (CA 125) at 273 U/mL, C-reactive protein (CRP) at 1.27 mg/dL, tuberculosis screening test (T-SPOT) positive, and anti-Mycobacterium avium-intracellulare complex (*MAC*) antibody positive (Table 1). An elevated erythrocyte sedimentation rate (ESR) of 30 mm/h was also observed. All other findings were within normal ranges. A plain chest radiograph showed opacities in the right middle and lower lung fields, and a granular shadow in the left lung apex (Figure 1).

**Table 1:** Laboratory findings at the first hospital visit Alb, albumin; ALT, alanine transaminase; AST, aspartate aminotransferase; BUN, blood urea nitrogen; CA 125, cancer antigen 125; Cl, chloride; Cr, creatinine; CRP, C-reactive protein; ESR, erythrocyte sedimentation rate; HGB, hemoglobin; HCT, hematocrit; K, potassium; LDH, lactate dehydrogenase; MAC, mycobacterium avium complex; Na, sodium; PCR, polymerase chain reaction; PLT, platelet; RBC, red blood cell; TP, total protein; T-SPOT, tuberculosis screening test; WBC, white blood cell count.

Laboratory investigation	Patient’s results	Reference range
WBC	5,100	3,300-8,600/μL
Neutrophils	64.4	41.2-69.7%
Lymphocytes	21.9	22.1-46.9%
Monocytes	11.2	4.1-9.6%
Eosinophils	1.9	0.0-3.5%
Basophils	0.6	0.0-1.1%
RBC	417×10^4^	386-492×10^4^/μL
HGB	12.6	11.6-14.8 g/dL
HCT	38.1	35.1-44.4%
PLT	32.4×10^4^	15.8-34.8×10^4^/μL
ESR	30	3-15 mm/h
TP	7.7	6.6-8.1 g/dL
Alb	4.4	4.1-5.1 g/dL
AST	16	13-30 IU/L
ALT	10	7-23 IU/L
LDH	164	124-222 IU/L
BUN	26.1	8.0-20.0 mg/dL
Cr	0.47	0.46-0.79 mg/dL
Na	140	138-145 mEq/L
K	4.6	3.6-4.8 mEq/L
Cl	106	101-108 mEq/L
CRP	1.27	0.00-0.14 mg/dL
CA 125	273	≤35 U/mL
T-SPOT	(+)	(-)
Anti-*MAC* antibody	(+)	(-)
Acid-fast test, sputum smear	(-)	(-)
PCR	(-)	(-)
Culture	(-)	(-)

**Figure 2:**
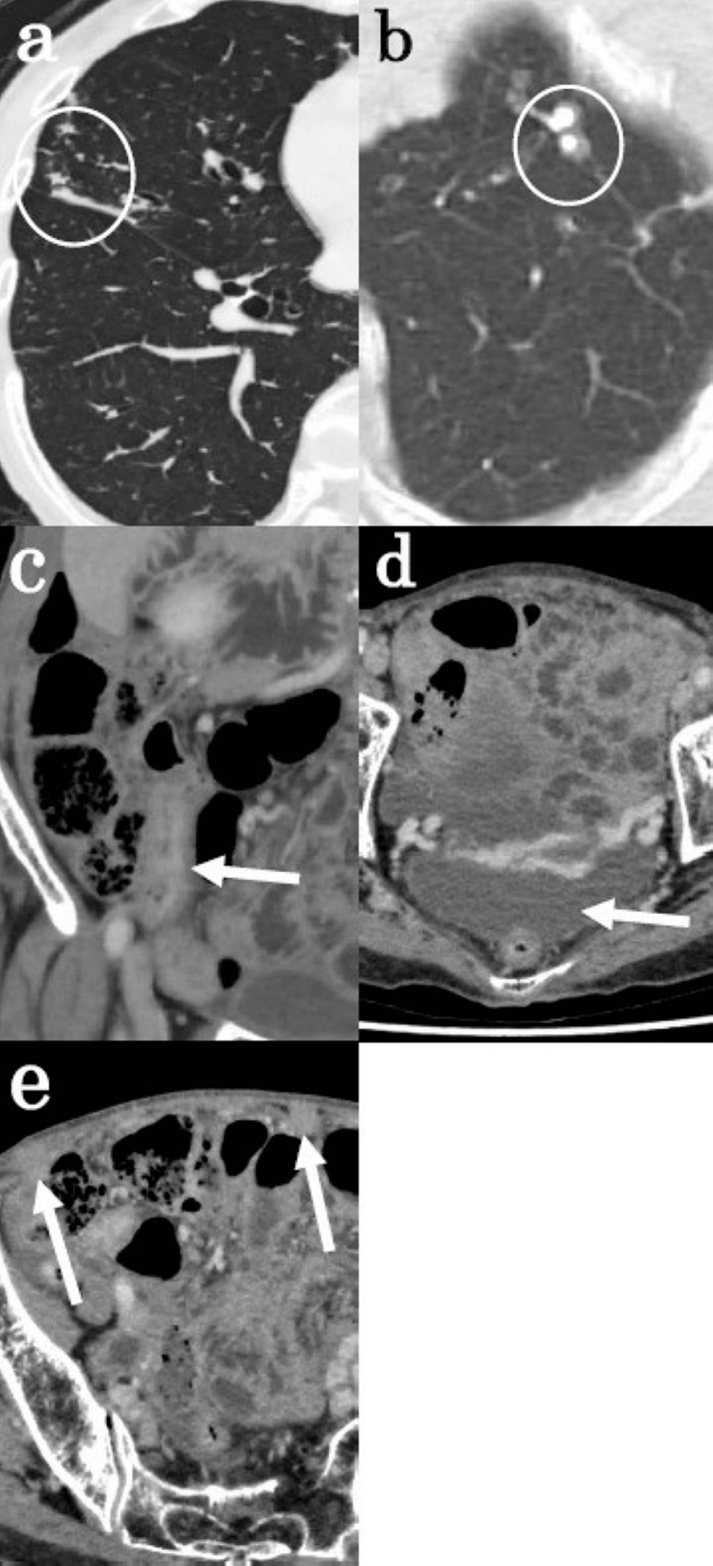
Contrast-enhanced CT scan taken at the first visit Contrast-enhanced CT scan taken at the first visit showing (a) a granular shadow in the right middle lung field; (b) calcified lesions in the left lung apex; (c) a 50-mm tumor in the ileocecal region that is darkly contrasted, suggestive of a malignant lesion; (d) ascites in the Douglas’ pouch; and (e) peritoneal thickening suggestive of carcinomatous peritonitis.

Contrast-enhanced computed tomography (CT) at the first visit also showed a granular shadow in the right middle lung field, calcified lesions in the left lung apex, a 50-mm darkly contrasted tumor in the ileocecal region that was suggestive of a malignant lesion, ascitic fluid in the pouch of Douglas, and peritoneal thickening that was suggestive of carcinomatous peritonitis (Figure 2).

**Figure 3:**
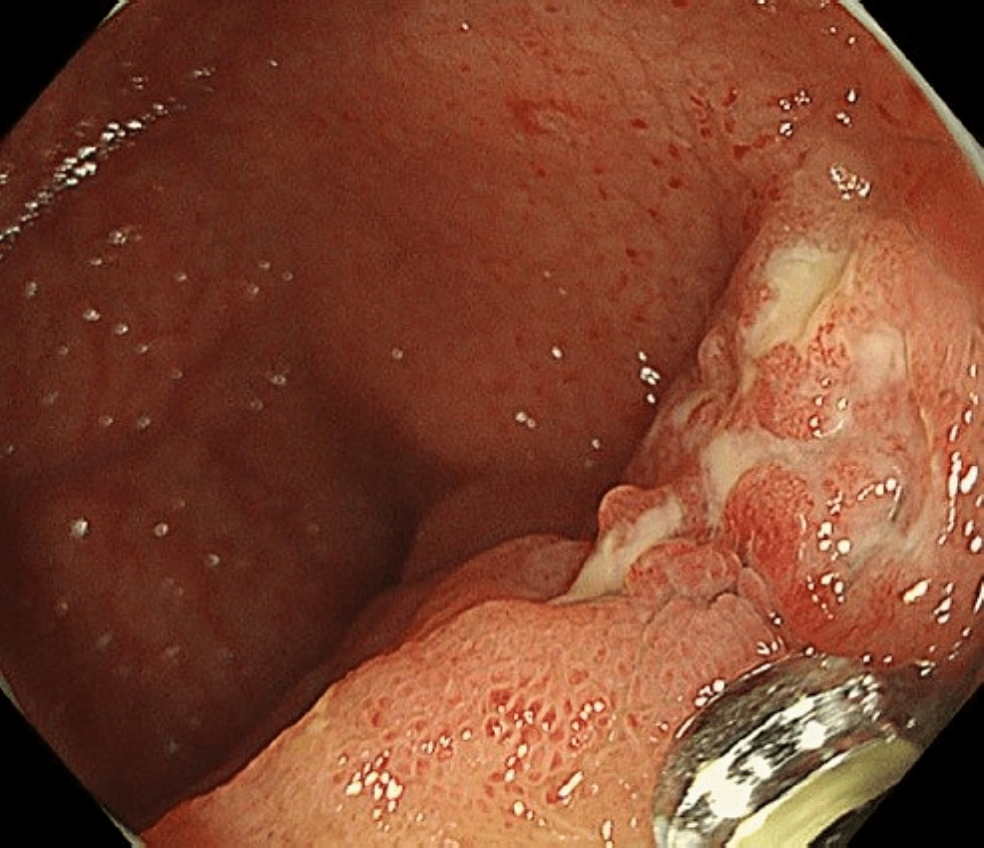
Colonoscopy Colonoscopy showing an ulcerative lesion in the terminal ileum.

Abdominal ultrasonography showed parietal peritoneal thickening. We did not identify acid-fast bacilli in her sputum after three consecutive days; however, her T-SPOT was positive. Exploratory laparoscopic surgery was scheduled in August; however, an acid-fast test and culture that were performed on samples obtained using colonoscopy from an ulcerative lesion in the ileum (Figure 3) turned out positive.

**Figure 4:**
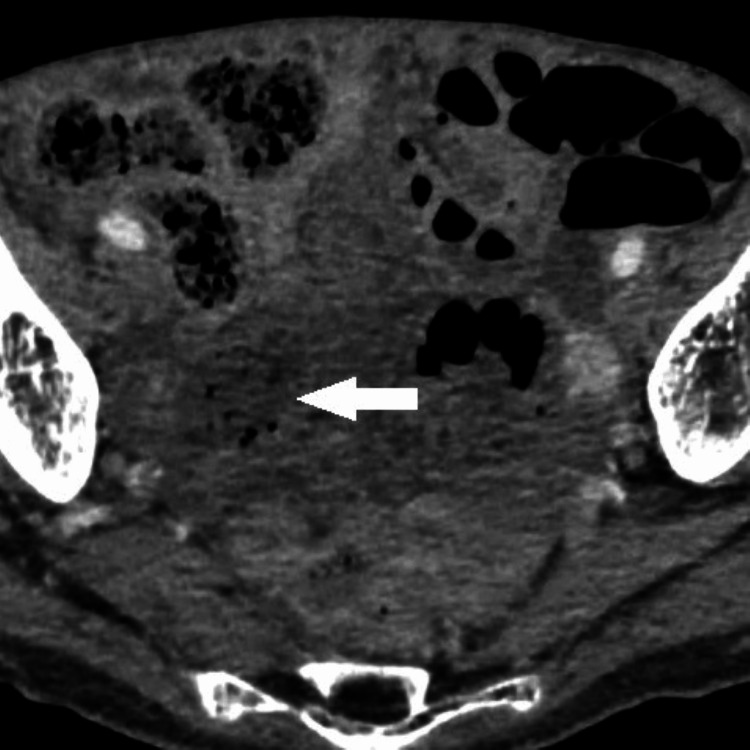
Contrast-enhanced CT scan taken at the visit for abdominal pain A contrast-enhanced CT scan taken at the visit for abdominal pain showed that the small intestinal walls are not enhanced. The patient was diagnosed with a strangulated small bowel obstruction.

Thus, the surgery was not performed. *Mycobacterium tuberculosis *complex was identified using polymerase chain reaction (PCR). We made a diagnosis of intestinal tuberculosis and commenced the administration of four anti-tuberculosis drugs: isoniazid (INH) 300 mg/day, rifampicin (RFP) 450 mg/day, ethambutol (EB) 750 mg/day, and pyrazinamide (PZA) 1.5 g/day. Two weeks after the initiation of therapy, abdominal ultrasonography showed improvement in the parietal peritoneal thickening and ascites. Four weeks after the initiation of therapy, further improvements were seen. However, in October, six weeks after the initiation of anti-tuberculosis therapy, she visited our hospital with complaints of abdominal pain. A contrast-enhanced CT scan showed unenhanced small intestinal walls (Figure 4).

**Figure 5:**
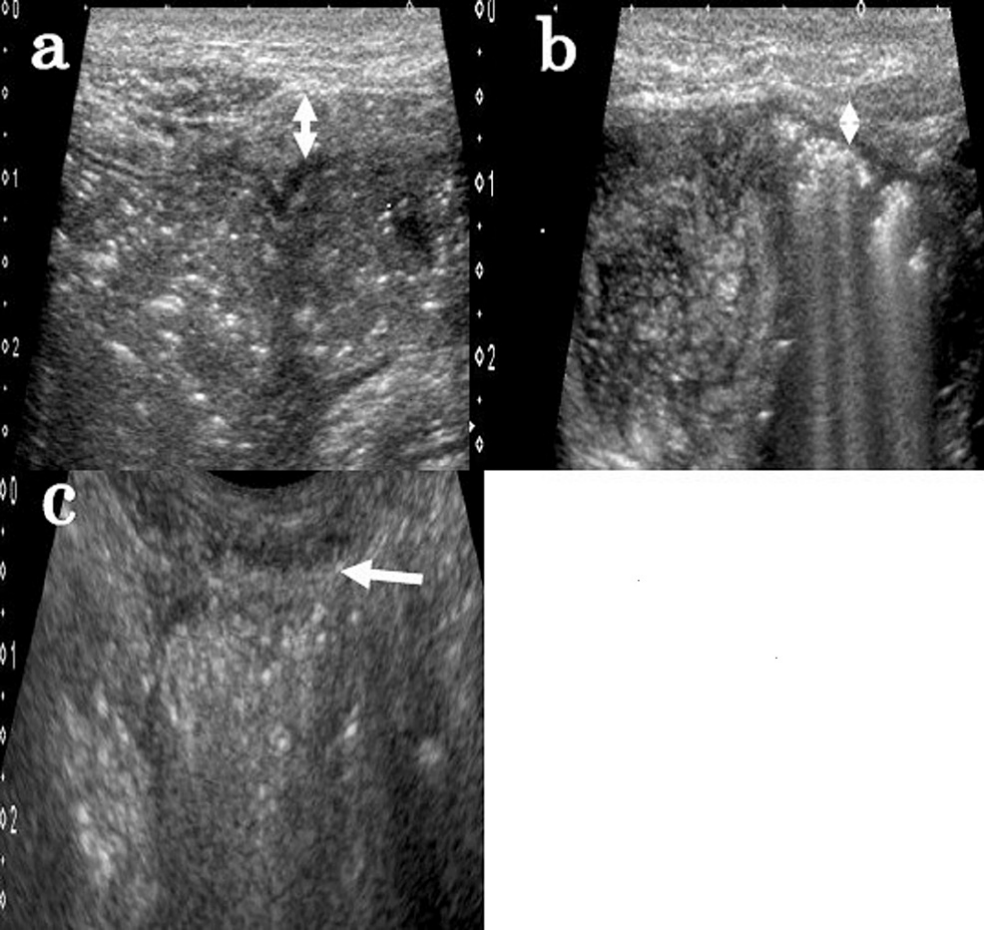
Abdominal ultrasonography Abdominal ultrasonography showing chronological improvement in the parietal peritoneal thickening at (a) the onset of treatment, (b) two weeks after the initiation of therapy, and (c) eight weeks after the initiation of therapy.

The patient was diagnosed with a strangulated small bowel obstruction. However, the strangulated small bowel obstruction resolved spontaneously without surgery, and she was discharged on the seventh day of hospitalization. One week after she was discharged, the parietal peritoneal thickening disappeared (Figure 5).

We tapered the four anti-tuberculosis drugs to two, INH 300 mg/day and RFP 450 mg/day, and completed the treatment in February 2022. Abdominal ultrasonography was performed every four weeks until the end of treatment, and no relapse was observed. At the end of the therapy, colonography revealed that the ulcerative lesion had become scarred, and no *Mycobacterium tuberculosis* was detected using the acid-fast test. No significant changes in her pulmonary lesions were observed at the end of therapy.

## Discussion

We identified two important clinical issues in this study. First, spontaneous resolution of strangulated small bowel obstruction can occur after starting anti-tuberculosis therapy. Second, abdominal ultrasonography is useful in evaluating the therapeutic effect of intestinal tuberculosis and tuberculous peritonitis.

First, spontaneous resolution of strangulated small bowel obstruction can occur after starting anti-tuberculosis drugs. Strangulated small bowel obstruction is a relatively common cause of an acute abdomen and requires urgent surgical treatment. Although surgery is often required, our patient’s strangulated small bowel obstruction resolved naturally, which caught our attention. The causes of strangulated small bowel obstruction are many, including primary volvulus, hernia, adhesion, bands, and intussusceptions [4]. Hernias and bands can spontaneously release an obstruction. The patient had no surgical history; however, bands may have formed during scarring due to the intestinal tuberculosis lesion. Anti-tuberculosis therapy can also promote fibrous changes. The main types of internal hernia that could lead to obstruction include paraduodenal, pericecal, foramen of Winslow, transmesenteric and transmesocolic, intersigmoid, and retroanastomotic hernias [5]. In addition, internal hernias, through a defect in the broad ligament of the uterus, can occur. These types of hernias could not be completely excluded as the cause of the strangulated small bowel obstruction because surgery was not performed. However, we considered that her strangulated small bowel obstruction may have been caused by bands, taking into account her course of treatment.

In this case, we could not diagnose tuberculous peritonitis bacteriologically. However, the patient’s symptoms, imaging findings, and inflammatory markers improved after the initiation of therapy; thus, we concluded that the patient’s peritonitis was tuberculous peritonitis. Moreover, a report showed that elevated serum CA 125 in patients with tuberculous peritonitis normalizes with the improvement of symptoms [6], which was also confirmed in this case. CA 125 is a useful marker of ovarian cancer. However, CA 125 is expressed in the coelomic epithelium, such as the pleura, peritoneum, and pericardium [7]. Thus, CA 125 is thought to drain into the ascitic fluid as a result of peritonitis, and inflammation in the area destroys the vessels, resulting in CA 125 to accumulate in high levels in the blood [8]. These two facts imply that the peritonitis, in this case, was tuberculous peritonitis.

Mycobacterial infections of the gastrointestinal tract occur through one of the following ways: (1) swallowing of infected sputum in a patient with active pulmonary disease; (2) hematogenous or lymphatic spread from a distant focus; (3) direct extension from a contiguous site; or (4) ingestion of milk products infected with *Mycobacterium bovis* [9,10]. Regarding this case, although the exact mechanism of tuberculosis remains unclear, hematogenous or lymphatic spread is the easiest to consider. However, the swallowing of infected sputum is not completely undeniable because her lung lesions were not diagnosed bacteriologically. The pulmonary lesions were thought to have been scarred; however, we recommend that bronchoscopic examination be performed for the detection of nontuberculous mycobacteria in cases where exacerbation of the imaging findings is observed.

Second, abdominal ultrasonography is useful in evaluating the therapeutic effects of the treatment of intestinal tuberculosis and tuberculous peritonitis. One study found that the abdominal ultrasonographic findings of 38 cases of tuberculous peritonitis revealed the involvement of the parietal peritoneum in 89.4%, ascites in 84.2%, anomalies of the greater omentum in 73.6%, and mesentery abnormalities in 63.1% of the patients [11]. In this case, all of these findings were confirmed at the onset of treatment and were consistent with the clinical diagnosis of tuberculous peritonitis. Ultrasonography is useful in observing multiple fine septations, which are characteristically found in tuberculous peritonitis, while computed tomography can detect peritoneal, mesenteric, and omental involvement [12-14]. These imaging modalities are complementary. We also confirmed chronological improvement using abdominal ultrasonography in a noninvasive manner.

## Conclusions

Spontaneous resolution of strangulated small bowel obstruction can occur after commencing anti-tuberculosis drugs. Abdominal ultrasonography is useful in evaluating the therapeutic effects of treatment in patients with intestinal tuberculosis and tuberculous peritonitis. Accordingly, appropriate diagnosis and management can prevent unnecessary surgical intervention. Further reports should be accumulated to determine whether the spontaneous resolution of strangulated small bowel obstruction is a more frequent occurrence and whether routine ultrasonography may be helpful in monitoring cases of intestinal tuberculosis.
